# Examining internal migration effects on short versus long interbirth intervals in Cotonou, Benin Republic

**DOI:** 10.1186/s12884-019-2529-7

**Published:** 2019-10-23

**Authors:** Boladé Hamed Banougnin

**Affiliations:** 1 Panafrican University, Life and Earth Sciences Institute (Including Health and Agriculture), University of Ibadan, PMB 5017, GPO, Ibadan, Nigeria; 2 Ecole Nationale de la Statistique, de la Planification et de la Démographie, Université de Parakou, Route de l’Okpara, B.P. 123, Parakou, Bénin

**Keywords:** Internal migration, Interbirth interval, Competing-risks, Cotonou, Benin Republic

## Abstract

**Background:**

The literature on migration-fertility relationship uses various measures of fertility, such as fertility rates, actual fertility and family size preferences. This study introduces a different measure—interbirth intervals over women’s reproductive years—to examine how internal migration is associated with short interbirth intervals (less than 24 months) and long interbirth intervals (greater than 60 months) in Cotonou, the largest city of Benin Republic.

**Methods:**

The paper uses primary data on 2852 live births to 1659 women aged 15–49 years from the 2018 Fertility and Migration Survey in Cotonou. Competing-risks models were fitted for the analysis.

**Results:**

Nineteen percent of live births were of short interbirth intervals and 16% were of long interbirth intervals. The prevalence of short interbirth intervals was higher among migrants who spent less than 5 years in Cotonou (29%) than among non-migrants (19%) and earlier migrants (18%). Non-migrants had the highest proportion of long interbirth intervals (19%). Within the first 5 years following the migration to Cotonou, migrants had higher subhazard ratio (SHR) of short interbirth intervals (SHR: 1.71, 95% CI: 1.33–2.21) and lower SHR of long interbirth intervals (SHR: 0.64, 95% CI: 0.47–0.87) than non-migrants. This association holds after controlling for socioeconomic characteristics—but with a slightly reduced gap between migrants who spent less than 5 years in Cotonou and non-migrants. Afterwards and irrespective of women’s socioeconomic backgrounds, migrants who spent 5 or more years in Cotonou and non-migrants had similar risks of short and long interbirth intervals. Finally, from 5 years of stay in Cotonou, migrants for reasons other than school or job were less likely to experience short interbirth intervals (SHR: 0.65, 95% CI: 0.46–0.98 for migrants who spent 5–10 years in Cotonou, and SHR: 0.74, 95% CI: 0.54–1.02 for migrants who spent more than 10 years in Cotonou) than non-migrants.

**Conclusion:**

Family planning programmes should mainly target migrants in the early years after their arrival in Cotonou. Moreover, non-migrants need to be sensitised on the adverse health outcomes of long interbirth intervals.

## Background

Like many sub-Saharan African (SSA) countries, Benin Republic is undergoing rapid urbanization [1]. The urbanization rate in the country increased from 34% in 1990 to 45% in 2013 [2]. The increasing urbanization is mainly driven by migration from rural to urban areas [3]. Internal migration—understood as a change in usual residence within a country—from rural to urban areas shifts the rural-urban balance of the population. Rural-urban migration in Benin Republic will make the country reach an urbanization rate of 61% by 2050 according to demographic projections of the United Nations [1]. Population specialists attributed part of the recent decline in fertility in SSA countries to the increased urbanization. In Benin Republic, they forecast a decrease in the total fertility rate from 4.9 children per woman in 2010–2015 to 3.8 in 2025–2030 [4]. As in many SSA countries, the pace of the decline in fertility rates in Benin Republic is not sufficient enough to sustain economic growth and reduce poverty [5, 6].

Although numerous studies have shown that urbanization leads to lower fertility in low- and middle-income countries (LMICs), there is still a need to understand how internal migration to cities alter fertility behaviours. Several works have concluded that the fertility of rural-urban migrants may be either different to, or the same as that of non-migrants in cities; but many of the conflicting conclusions reflect differences in study designs, analytical methods, definitions of migrants, and fertility measures used [7–10]. Among other matters, the questions about the timing of changes in migrants’ fertility behaviours, and the way in which rural-urban migration affects these changes are sorely lacking. This lack of knowledge makes the assessment of the relationship between migration and fertility difficult.

This study investigates the relationship between internal migration and fertility, using interbirth interval (IBI)—time between two successive live births—as fertility indicator. Using IBI as fertility indicator to analyse the migration-fertility relationship is relevant for both population and reproductive health researchers [11]. Interbirth interval is closely related to maternal and child health as well as the population dynamic. Short and long interbirth intervals have a critical influence on perinatal and maternal outcomes. Research has linked short IBIs (of less than 24 months) to increased risks for preterm birth, low-birth-weight or small for gestational age, labour dystocia, and maternal morbidity and mortality [12–14]. Short IBIs are also associated with high risks of premature rupture of membranes, third-trimester bleeding, anaemia, and puerperal endometritis which place women at greater risk of haemorrhage—the primary cause of maternal death in LMICs [13, 15]. However, relatively longer IBIs (of greater than 60 months) are associated with higher risks of preeclampsia and dystocia [16, 17]. In Benin Republic, the median interbirth interval increased from 35 months in 2006 to 36 months in 2012 [12]. In Cotonou—Benin Republic’s largest city, it decreased from 39 to 38 months during the same period [12].

Studies have identified migration as a life-changing event affecting migrants’ fertility behaviours [8, 18]. Research suggests four partly complementary, partly contradictory theoretical approaches for explaining the fertility of migrants: the socialization, adaptation, selection, and disruption hypotheses [8, 19]. The migration flows of concern in this study are those from rural areas and small cities to large cities. It is worth mentioning that the largest cities in SSA countries usually have the lowest fertility rates and are always considered as lower-fertility settings. The socialization hypothesis suggests that the fertility behaviours of migrants primarily reflect the fertility preferences dominant in their place of origin. Thereafter, migrants might exhibit the fertility patterns of the host society after a generation at least has elapsed [8, 20]. In contrast, the adaptation hypothesis proposes that the fertility of migrants may gradually converge to that of non-migrants at the destination environment. Testing the socialization hypothesis requires specific timely data about families and life course trajectories of individuals [20]. The adaptation effect operates over time; that is with the duration of residence, and can only present if there is a difference between the fertility of non-migrants and the fertility of migrants just after their arrival [8, 19]. In this work, migrants are expected to be more likely to have short IBIs and less likely to have long IBIs than non-migrants just after their arrival in Cotonou (Hypothesis 1a) and thereafter—with time passing, to exhibit similar IBIs as non-migrants in Cotonou (Hypothesis 1b).

The selection hypothesis argues that rural-urban migrants are self-selected groups with relatively lower fertility preferences that prepare them to adapt to lower urban fertility [8, 19, 21]. Migrants in cities are generally well-educated, often have well-paying job opportunities, which favour delays in childbearing [18, 22]. Migrants in Cotonou are likely to have similar (high) socioeconomic characteristics to non-migrants in Cotonou. In consequence, migrants and non-migrants in Cotonou may, net of socioeconomic characteristics, have similar risks of experiencing short and long interbirth intervals (Hypothesis 2). Finally, the disruption hypothesis postulates that during the period immediately following migration, migrants exhibit particularly low levels of fertility due to the disruptive factors associated with the migration process [7, 23–25]. This hypothesis implies that recent migrants from high to low fertility settings will have lower fertility compared to earlier migrants from high to low fertility settings. It also implies that recent migrants will have lower fertility compared to non-migrants for the period immediately following and possibly preceding migration irrespective of the migration flows. The disruption theory specifically suggests that certain migrations (migration for reasons such as job or school) lead to delays in childbearing [7, 23–25]. In line with the disruption theory, recent migrants for school or job reason are expected to be less likely to experience shorter interbirth intervals than non-migrants and earlier migrants in Cotonou (Hypothesis 3).

Few studies exist on short and long interbirth intervals in SSA countries [26] and even none on their association with migration. This paper examines the extent of IBIs differentials between migrant and non-migrant women, as well as between categories of migrants as defined by type (for job or school reason or not) and recency of move. The study focuses on migration from rural areas and small cities of Benin Republic to Cotonou. It uses data from the 2018 Cotonou Fertility and Migration Survey (FMS) to give insight to the adaptation, selection and disruption hypotheses of the migration-fertility relationship. In so doing, it aims to better identify the categories of women at higher risks, in order to spot the target populations for appropriate actions.

## Methodology

### Data

The paper uses primary data collected during the 2018 Fertility and Migration Survey (FMS) in Cotonou. The overarching goal of the 2018 Cotonou FMS was to provide data on migrants’ fertility behaviours. Two questionnaires (available either in Additional file 1 and Additional file 2 or at https://github.com/bolade1227/fms) were implemented in the 2018 Cotonou FMS: household and women aged 15–49 years. The households were selected using a stratified two-stage cluster design. At the first stage, 56 enumeration areas (EAs) were drawn from the 2013 Benin Republic population and housing census sampling frame. Then, 35 households were randomly selected per EA. Finally, residents in the selected households were enumerated and all eligible women aged 15–49 years were interviewed.

The final sample consisted of 1913 households and 1949 women of reproductive age.^1^ The questionnaires obtained information on women’s migration and birth histories, their fertility preferences, family planning practices, and other socio- economic and demographic variables. Information on migration included the respondent’s previous place of residence, duration of residence in Cotonou, and principal reason for residing in Cotonou.

Live births to migrant women that had not occurred in Cotonou were excluded from the sample; this yielded a sample of 3336 live births among 1226 women (with the exception of international migrant women). From among these live births, the sample was then restricted to 2110 live births with information on interbirth intervals. The weighted sample was 2852 live births among 1659 women aged 15–49 years.^2^

### Data collection and management

Data were collected using CSPro’s Computer Assisted Personal Interviewing (CAPI) application developed and deployed on Android devices through CSEntry (see www.csprousers.org for more details on CSPro). Data collectors were employed and trained on the use of CAPI for data collection. The household questionnaire and women’s questionnaire were designed in French and translated by the enumerator in any local languages where required. Verbal informed consent was sought by the enumerator reading a prescribed statement to the respondent (aged 15–49 years) and recording in the questionnaire whether or not the respondent consented or provided assent on behalf of minors (under 16). Then, the enumerator signs her name attesting to the fact that she read the consent statement to the respondent. The ethics committee approved this procedure. Thereafter, the interview was privately held after the study participant consented to participate in the study. Data were then transcribed back to English for cleaning, screening, and analysis.

### Measures

The study examined interbirth intervals over women’s reproductive years. Interbirth interval was calculated in months and defined as the time between the current live birth and the following live birth, counting twins as one live birth. The reason of choosing interbirth interval as measure of spacing rather than interpregnancy interval is two-fold. First, the individual female questionnaire did not record pregnancies history. Retrospective data on pregnancies may be more biased by errors due to memory lapses than those on live births, especially when the period of analysis is too long (e.g., over women’s reproductive years). Particularly, in SSA countries where the illiteracy rate is still relatively high, women are more likely to forget a date of pregnancy than a date of childbirth. Second, interbirth interval remains an appropriate measure of spacing when considering the effect of competition or disease transmission from another young child in the family [28].

As in previous studies, interbirth intervals of less than 24 months were categorized as “short”, 24–60 months as “intermediate”, and greater than 60 months as “long”. Both short and long IBIs are considered as more risky than intermediate IBIs [12].

The main independent variable of the study is the migration status. Drawing on the work of Garcia et al. [29], information on the previous place of residence and duration of residence of women in Cotonou was used to define migration. Migrants in this study are women who were living in Cotonou at least 6 months before the survey and who were previously residing somewhere else within Benin Republic. Migrants were then classified into three groups: those who spent less than 5 years in Cotonou, 5–10 years in Cotonou, and more than 10 years in Cotonou.

The literature often cites several socio- economic and demographic variables—education among others—as greatly associated with interbirth intervals as well [15, 30]. Furthermore, scholars who investigated the migration-fertility relationship have repeatedly used socio- economic and demographic variables in order to control for selective migration [19, 22, 23]. The socio- economic and demographic variables used in this study included: religion, education, employment, ideal number of children, modern contraceptive use, and marital status. The baseline control variables for the analysis are: previous number of child deaths, mother’s age at birth index, birth index, having children of both sexes at birth index or not. Finally, interaction terms between reason for migration to Cotonou and migration status were created.

### Statistical analysis

Event-history analysis was employed to examine the association between migration and interbirth intervals over women’s reproductive years. The analysis time (in months) is the duration between two successive live births. The IBI variable records the type of event observed: 0 for intermediate IBI (24–60 months of interval), 1 for short IBI, and 2 for long IBI.

Competing-risks models that treat a short IBI as the event of interest and a long IBI as the competing event, and vice-versa were fitted. During her reproductive years, a woman who did not experience short IBI may experience long IBI and vice-versa—considering intermediate IBI as less risky for maternal and child health [17]. In this case, using standard survival analysis like Cox proportional hazard (PH) model presents a number of limitations [31–33]. As a remedy, Fine and Gray [34] proposed cumulative incidence function (CIF) based PH model to analyse survival data arising from a competing risk setup. They then specified a model for the hazard of the subdistribution of the different events at time *t* as

1 $$ {\overline{h}}^{(k)}(t)=\underset{dt\to 0}{\lim }P\left[\left.\left(t<T\le t+ dt\&{Y}^{(k)}(t)=1\right)\right|T>t\kern0.5em or\kern0.5em \left(T\le t\kern0.5em \&\kern0.5em {Y}^{(k)}(t)=0\right)\right]/ dt $$

where *Y*^(*k*)^(*t*) is an indicator function with *Y*^(*k*)^(*t*) = 1 if the event of type *k* (here 1 for short IBI and 2 for long IBI) occurs.

The advantage of modelling the subdistribution hazard, or subhazard, is that one can calculate the CIF from it;

2 $$ {CIF}^{(k)}(t)=1-\exp \left\{-{\overline{H}}^{(k)}(t)\right\} $$

where ${\overline{H}}^{(k)}(t)={\int}_0^t{\overline{h}}^{(k)}(t)\kern0.5em dt$ is the cumulative subhazard.

Competing-risks regression performed in this manner is similar to Cox regression.

The model is semiparametric in that the baseline subhazard ${{\overline{h}}_0}^{(k)}\kern0.5em (t)$ (that for covariates set to zero) is left unspecified, while the effect of the covariates *X* are assumed to be proportional:

3 $$ {\overline{h}}^{(k)}\left(t\left|X\right.\right)={{\overline{h}}_0}^{(k)}\kern0.5em (t)\exp \kern0.5em \left( X\beta \right) $$

where *β* is the vector of coefficients.

For each woman *i*, the subhazard at time *t* is assumed to be

4 $$ {\overline{h}}^{(k)}\left(t\left|X\right.\right)={{\overline{h}}_0}^{(k)}\kern0.5em \left(\beta \hbox{'}{X}_i\right) $$

The exponential of coefficients produces subhazard ratios (SHRs). A positive (respectively negative) coefficient means that the effect of increasing that covariate is to increase (respectively decrease) the subhazard and therefore increase (respectively decrease) the CIF across the board. The competing-risks models—all models being initially controlled for previous number of child deaths, mother’s age at birth index, birth index, having children of both sexes at birth index or not—used for this study can be summarized as follows.

- Model 1, 1b: Show the relationship between migration and short versus long IBIs. This model gives insight to the adaptation hypothesis (Hypotheses 1a and 1b).
- Model 2, 2b: Model 1, 1b plus six socio- economic and demographic variables—education, employment status, ideal number of children, modern contraceptive use, religion, and marital status. This model enables to test the selection hypothesis (Hypothesis 2).
- Model 3, 3b: Model 2, 2b with migration status being replaced by interaction terms (migration * reason for migration to Cotonou). Model 3, 3b allows to test the disruption hypothesis (Hypothesis 3).

Statistical analysis was performed using STATA version 13.1 (StataCorp, TX, USA). SHRs were calculated along with 95% confidence intervals.

## Results

### Characteristics of live births

**Fig. 1:**
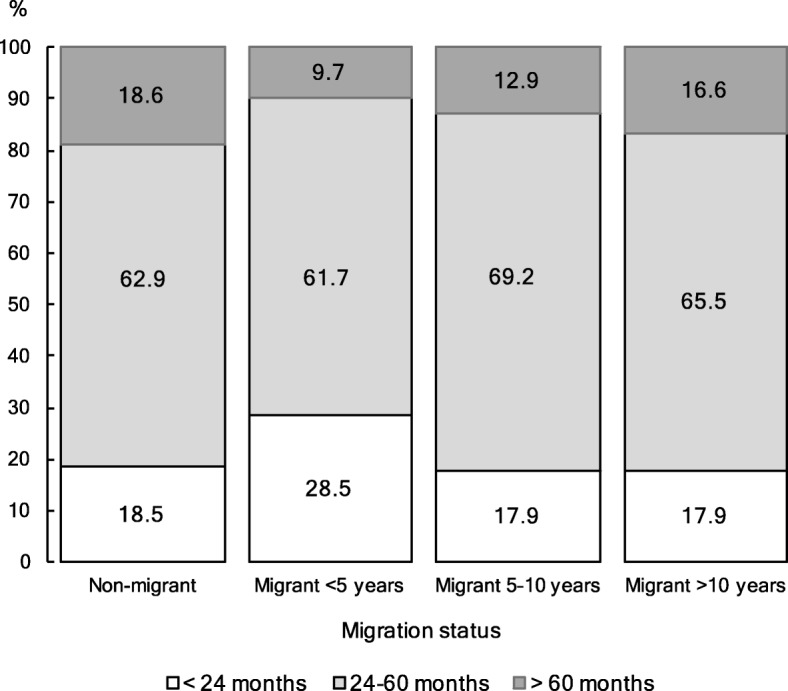
Percentage distribution of live births among Cotonou women aged 15–49, by interbirth interval and according to migration status

Nineteen percent of live births occurred after intervals of less than 24 months, 65% occurred after intervals of 24–60 months and 16% occurred after intervals of greater than 60 months. The prevalence of short IBIs was higher among migrant women who spent less than 5 years in Cotonou (29%) than among non-migrant women (19%) and earlier migrant women (18%). Conversely, 10% of births among the most recent migrant women (who spent less than 5 years in Cotonou) were of long interval against 19% for non-migrant women. The proportion of long IBIs was 13% among migrant women who have been residing in Cotonou for 5–10 years and 17% among the earliest migrant women (who had resided in Cotonou for more than 10 years) (Fig. 1).

Overall, 37% of live births were among non-migrant women, 10% among most recent migrant women, 17% among migrant women who spent 5–10 years in Cotonou, and 36% among the earliest migrant women (Table 1). The majority of live births were among women who were employed (61%), reported 4 or more children as ideal number of children (73%), were non-Muslims (85%), and were married (86%). On average, maternal age at birth index was 24 years. The mean number of live births and child deaths at birth index was 2.1 and 0.1 respectively.

**Table 1:** Distribution of live births, by selected characteristics and according to interbirth interval

Variable	N	%/Mean (SD)	Interbirth interval		
			< 24 months	24–60 months	> 60 months
Overall	2852	100.0	19.2	64.8	16
Migration status ^***^					
Non-migrant	1053	36.9	18.5	62.9	18.6
Migrant					
< 5 years in Cotonou	289	10.1	28.5	61.7	9.7
5–10 years in Cotonou	497	17.4	17.9	69.2	12.9
> 10 years in Cotonou	1014	35.5	17.9	65.5	16.6
Migrated for job or school reason^(ns)^					
Yes	358	12.5	20.7	64.1	15.3
No	2494	87.5	19.0	64.9	16.1
Education ^***^					
No education	1110	38.9	21.3	64.7	14.0
Primary	1001	35.1	18.8	65.8	15.4
Secondary or higher	741	26.0	16.5	63.7	19.8
Employment ^**^					
Employed	1732	60.7	17.6	65.9	16.6
Unemployed	1121	39.3	21.7	63.2	15.2
Ideal number of children ^***^					
0–3 children	777	27.2	17.3	63.4	19.3
> 3 children	2076	72.8	19.9	65.3	14.8
Modern contraceptive use ^(ns)^					
User	416	14.6	18.4	68.4	13.2
Non-user	2436	85.4	19.3	64.2	16.5
Religion ^*^					
Muslim	438	15.4	22.7	63.5	13.8
Non-Muslim	2414	84.6	18.5	65.0	16.4
Marital status ^***^					
Married	2446	85.7	17.9	66.0	16.1
Unmarried	407	14.3	27.0	57.4	15.6
Had children of box sexes ^(ns)^					
Yes	483	16.9	19.8	61.9	18.2
No	2370	83.1	19.1	65.4	15.6
Maternal age at birth index^(a)^		24.0 (0.1)	24.3 (0.2)	23.9 (0.1)	23.7 (0.3)
Previous number of live births^(a)^		2.1 (0.0)	2.2 (0.1)	2.1 (0.0)	2.1 (0.1)
Previous number of child deaths^(a)^		0.1 (0.0)	0.2 (0.0)	0.1 (0.0)	0.1 (0.0)

Note. % = percentage; SD = standard deviation; N = weighted number of observations; ns = non-significant; Chi-square statistic *** *p* < 0.01, ** *p* < 0.05, * *p* < 0.1; (a) Column means vary significantly at 5% significance level

### Multivariate analysis

Table 2 presents subhazard ratios (SHRs) and 95% confidence interval of SHRs for migration status and other covariates obtained from Fine and Gray [34] competing-risks survival regression models for short and long IBIs.

**Table 2:** Subhazard ratio (SHR) obtained from the Fine-Gray models for short and long IBIs

	Short interbirth intervals			Long interbirth intervals		
	Model 1	Model 2	Model 3	Model 1b	Model 2b	Model 3b
Migration						
Non-migrant (Ref.)	1	1		1	1	
Migrant						
< 5 years in Cotonou	1.71*** (1.33–2.21)	1.60*** (1.23–2.08)		0.64*** (0.47–0.87)	0.67** (0.50–0.91)	
5–10n years in Cotonou	0.97 (0.76–1.24)	0.90 (0.69–1.16)		0.95 (0.78–1.16)	0.98 (0.80–1.21)	
> 10 years in Cotonou	0.99 (0.81–1.21)	0.97 (0.78–1.19)		1.02 (0.89–1.18)	1.06 (0.92–1.23)	
Migration * Reason for migration						
Non-migrant (Ref.)			1			1
In-migrant						
< 5 years in Cotonou						
For job or school reason			1.52** (1.03–2.26)			0.67* (0.43–1.06)
Neither for job nor for school reason			1.59**(1.11–2.28)			0.67* (0.45–1.01)
5–10 years in Cotonou						
For job or school reason			1.13 (0.81–1.59)			1.03 (0.80–1.31)
Neither for job nor for school reason			0.67** (0.46–0.98)			0.89 (0.60–1.31)
> 10 years in Cotonou						
For job or school reason			1.16 (0.88–1.53)			1.03 (0.85–1.25)
Neither for job nor for school reason			0.74* (0.54–1.02)			1.12 (0.89–1.40)
Education						
No education (Ref.)		1	1		1	1
Primary		0.81** (0.66–1.00)	1.58*** (1.20–2.09)		1.08 (0.91–1.27)	0.85 (0.69–1.05)
Secondary or higher		0.71*** (0.57–0.90)	0.80** (0.65–0.99)		1.15* (0.98–1.36)	1.07 (0.91–1.27)
Employment						
Employed (Ref.)		1	1		1	1
Unemployed		1.26*** (1.07–1.50)	0.71*** (0.56–0.90)		0.86** (0.75–0.99)	1.15* (0.98–1.36)
Ideal number of children						
1–3 children (Ref.)	1	1		1		1
> 3 children	1.24** (1.01–1.52)	1.25** (1.02–1.54)		0.83*** (0.72–0.95)		0.82*** (0.71–0.95)
Modern contraceptive use						
User	1.02 (0.80–1.30)	1.00 (0.78–1.28)		0.97 (0.79–1.19)		0.97 (0.79–1.19)
Non-user (Ref.)	1	1		1		1
Religion						
Muslim	1.31** (1.04–1.65)	1.33** (1.06–1.67)		0.91 (0.75–1.10)		0.91 (0.75–1.11)
Non-Muslim (Ref.)	1	1		1		1
Marital status						
Married (Ref.)	1	1		1		1
Unmarried	1.70*** (1.37–2.12)	1.69*** (1.36–2.11)		0.69*** (0.58–0.83)		0.69*** (0.58–0.83)
Mother’s age at birth index	1.01 (0.99–1.03)	1.03*** (1.01–1.05)	1.03*** (1.01–1.05)	1.02*** (1.01–1.04)	1.01 (1.00–1.03)	1.01 (1.00–1.03)
Had children of both sexes						
Yes	1.06 (0.76–1.47)	1.06 (0.76–1.47)	1.05 (0.76–1.46)	0.91 (0.71–1.16)	0.93 (0.72–1.20)	0.93 (0.73–1.20)
No (Ref.)	1	1	1	1	1	1
Previous number of live births	1.01 (0.90–1.12)	0.95 (0.86–1.05)	0.94 (0.85–1.04)	0.97 (0.88–1.06)	1.01 (0.92–1.12)	1.01 (0.92–1.12)
Previous number of child deaths	1.18* (0.98–1.43)	1.20* (1.00–1.44)	1.19* (0.99–1.42)	0.83* (0.68–1.02)	0.83* (0.67–1.02)	0.82* (0.67–1.02)
Observations	2852	2852	2852	2852	2852	2852

***Note.*** 95% confidence interval for SHR in parentheses; Ref. = Reference category; *** *p* < 0.01, ** *p* < 0.05, * *p* < 0.1

Findings show that there are significant associations between internal migration to Cotonou and short versus long interbirth intervals. Migrant women who have been residing in Cotonou for less than 5 years have higher hazard rates of experiencing short IBIs (SHR: 1.71, 95% CI: 1.33–2.21) and lower hazard rates of experiencing long IBIs (SHR: 0.64, 95% CI: 0.47–0.87) than non-migrant women in Cotonou (Table 2, Model 1, 1b). However, the hazard rates of experiencing short and long IBIs do not differ significantly between earlier migrant women (who have been living in Cotonou for 5 years or more) and non-migrant women. Thus, migrant women’s and non-migrant women’s birth spacing practices converge over time, which is consistent with the gradual adaptation hypothesis (Hypothesis 1a and Hypothesis 1b).

After adjusting for socio- economic and demographic variables (Table 2, Model 2, 2b), the association observed between migration and IBIs in Model 1, 1b in Table 2 remains unchanged. But the gap in the hazard of experiencing short and long IBIs between migrant women who spent less than 5 years in Cotonou and non-migrant women in Cotonou has slightly decreased. Migrant women who had stayed in Cotonou for 5 years or more and non-migrant women in Cotonou still do not differ significantly concerning birth spacing practices. Migrant women who spent less than 5 years in Cotonou have a 60% higher risk of short IBIs (SHR: 1.60, 95% CI: 1.23–2.08) and a 33% lower risk of long IBIs (SHR: 0.67, 95% CI: 0.50–0.91), as compared to non-migrant women. The gradual adaptation effect persists, and to a lesser, the positive selection on less risky IBIs.

Finally, migration status was substituted for interaction terms between migration status and reason for migration in Model 3, 3b in Table 2. Hypothesis 3 posited that recent migrant women for job or school reason would have lower risk of shorter interbirth intervals as compared with non-migrant and earlier migrant women. The results first reveal that, concerning the experience of short IBIs, there is a delay in the disruptive effect, with women who migrated for reasons other than job or school having the lowest risks. In comparison with non-migrant women, migrant women have higher risk of short IBIs within the first 5 years following the migration, with the risk for the ones for job or school reason being lower than for the ones for reasons other than job or school (SHR: 1.52, 95% CI: 1.03–2.26 and SHR: 1.59, 95% CI: 1.11–2.28 respectively). Thereafter, from 5 years of stay in Cotonou, the hazard of short IBIs becomes lower among women who migrate for reasons other than job or school (SHR: 0.67, 95% CI: 0.46–0.98 for the ones who spent 5–10 years in Cotonou, and SHR: 0.74, 95% CI: 0.54–1.02 for the ones who spent more than 10 years in Cotonou) than among non-migrant women. Regarding the experience of long interbirth interval, significant differences are observed between only migrant women who stayed in Cotonou for less than 5 years (regardless of the reason for migration) and non-migrant women. Migrant women who spent less than 5 years in Cotonou are 33% less likely to experience long IBIs (SHR: 0.67, 95% CI: 0.43–1.06 if the reason for migration is school or job, and SHR: 0.67, 95% CI: 0.45–1.01 otherwise) than non-migrant women.

Women’s socio- economic and demographic characteristics are associated with interbirth interval, with unexpected changes from Model 2, 2b to Model 3, 3b (in Table 2). Findings from Table 2, Model 2, 2b show that unemployed women are more likely to experience short IBIs and less likely to experience long IBIs than employed women. After taking into account the reason for migration (Table 2, Model 3, 3b), women’s employment status is still significantly associated with short and long IBIs, but in reverse direction. The hazard rate changed from 1.26 (95% CI: 1.07–1.50) in Model 2 to 0.71 (95% CI: 0.56–0.90) in Model 3 for short IBI, and from 0.86 (95% CI: 0.75–0.99) in Model 2b to 1.15 (95% CI: 0.98–1.36) in Model 3b for long IBIs. Also, compared to women with no education, the hazard of short IBI shifted from a 19% lower risk in Model 2 to a 58% higher risk in Model 3 for women with primary education (SHR: 0.81, 95% CI: 0.66–1.00 in Model 2, and SHR: 1.58, 95% CI: 1.20–2.09 in Model 3). Women with secondary or higher education remain less likely to have short IBIs (Model 2 and Model 3) and more likely to have long IBIs (Model 2b only) than their peers with no education. Other variables such as the ideal number of children, religion, marital status, mother’s age at birth index, previous number of child deaths are associated with IBI as well. Reporting an ideal number of children of more than 3, being unmarried, and being Muslim are significantly associated with higher hazards of short IBIs. The higher the previous number of child deaths, the higher the hazard of short IBIs and the lower the hazard of long IBIs. The association between mother’s age at birth index and IBIs is weak.

## Discussion

This study uses data from the 2018 Cotonou FMS to examine how internal migration is associated with interbirth intervals in Cotonou, Benin Republic. The study particularly attempts to analyse this association through the adaptation, selection and disruption hypotheses. The results confirm the adaptation hypothesis, indicate a delay in the disruptive effect, and find some weak evidence to support the selection hypothesis. In addition, contrary to what was expected, the relatively long-term disruption effect happened within the group of women who migrated for reasons other than school or job. Other covariates such education, employment status, ideal number of children, religion, marital status, mother’s age at birth index, and previous number of child deaths are significantly associated with short and long IBIs. However, the way education and employment status are associated with IBIs changes when taking into account the interaction between migration and reason for migration.

First of all, findings reveal that migrant women’s birth spacing practices gradually converge to those of non-migrant women. In a relatively short-term (less than 5 years of stay in Cotonou), migrant women are at a higher risk of experiencing short IBIs and at a lower risk of experiencing long IBIs as compared to non-migrant women in Cotonou. Then, as time goes by, migrants tend to acquire the birth spacing patterns of non-migrants. Earlier migrant women and non-migrant women do not differ significantly in terms of birth spacing practices. The adaptation model depicted in this study is therefore gradual rather than abrupt. This finding is in line with previous research on the relationship between family planning and migration in LMICs [35, 36], which found in Guatemala and Kenya that migrants’ adaptation to contraceptive practices in the host society occurs over time. Indeed, the adaptation process itself works through the diffusion of the new environment’s contraceptive and fertility practices from natives to migrants in the host community [36, 37]. In urban areas especially, regular and free access to mass media facilitates the adaptation process. However, the process of knowledge accumulation among migrants takes time as the latter learn more about their new environment and expand their range of social contact over time [38].

After adjusting for women’s socioeconomic backgrounds (Table 2, Model 2, 2b), the hazard of short (respectively long) IBIs remains higher (respectively lower) for migrant women. But the gap in the hazard between migrant and non-migrant women decreased slightly. Thus, the analysis provides limited evidence to support the selection hypothesis. This conclusion differs from that of the majority of studies on migration-fertility relationship [8, 18, 19, 22]. Goldstein and Tirasawat [7] are among the few authors whose work provided limited evidence to support the selection hypothesis. Examining the fertility of migrants to urban places in Thailand, their findings lent strong support to the disruption hypothesis but provided weak evidence to support the selection effect in mobility to urban areas. The fact that the results from this study hardly support the selection hypothesis may be due to the absence of other socioeconomic variables that are specific to migrant women in Cotonou. Adding some economic-related variables to the model may bring out more evidence supporting the selection hypothesis. Contraceptive failure must be considered as a possible explanation. Two recent studies carried out by Banougnin and colleagues [39, 40] in Cotonou showed that the risk of contraceptive failure was higher among recent migrant women than among non-migrant women. Also, the proportion of migrants in Cotonou with high socioeconomic characteristics might be lower than expected. In future research, scholars might investigate specific socio- economic and demographic characteristics of migrants that favour lower risks of short and long interbirth intervals.

One key finding from this analysis is the striking role that the reason for migration plays in the directional change of the association between some covariates (migration, education, and employment status) and interbirth intervals. The lower hazard of short IBIs of long-term compared with most recent migrant women suggests several possible relations. Here, it is not the migration process itself that is disruptive of fertility—as described in the literature of the analysis of the relationship between migration and fertility [7, 8, 24], but probably the living conditions following the migration to Cotonou. There may be reasons for the disruptive character of the living conditions following the migration to Cotonou. Being a migrant and securing a first job in large cities itself may be sufficiently disturbing from a socio-psychological perspective as to interfere with the psychological capacity to conceive and bear children [41]. Furthermore, some women may have shortened interbirth intervals soon after arriving in Cotonou, and thereafter started delaying childbirth due to either hard living conditions or new job’s constraints. Further research should assess how education and employment status are associated with migrants’ fertility and according to the reason for migration.

This study contributes to the existing literature on migration-fertility relationship. It is a refreshing departure from other migration-fertility studies that focus almost exclusively on usual fertility indicators—cumulative fertility among others. Examining the relationship between migration and fertility using interbirth interval as fertility indicator is of interest for both demographers and reproductive health researchers. For demographers, the analysis of interbirth intervals is a more susceptible method for measuring fertility as compared to other methods [11, 26, 42]. Reproductive health researchers are interested in analysing interbirth intervals because of their association with adverse maternal and child health outcomes. The set of variables extracted from the 2018 Cotonou FMS data (migration status, duration of residence in Cotonou, and reason for migration to Cotonou) is sufficient and quite effective in interpreting the data.

The study presents some limitations. First, most of the covariates used in the models are time-invariant while the dependent variable is time-variant. Using longitudinal or event-history data offers possibilities of addressing this limitation. Second, when analysing infant and child mortality or inter-outcome related to a pregnancy, the use of interbirth interval as measure of spacing constitutes a limitation as it is biased by the gestational length of the second pregnancy. In this case, another alternative might be collecting data on births and pregnancies during a short calendar period.

## Conclusions

Future research on how migration is associated with short and long interbirth intervals, and their interaction with education and employment status may build on this work in several ways. First, using longitudinal data in which information about pregnancies and live births are collected would reduce errors due to memory lapses. The other alternative would be the use of event-history data recording pregnancies and live births during a short calendar period. Second, incorporating additional details on the reason for migration and the job episodes as well as education could help to assess the disruption and selection hypotheses with more precision. Finally, the results suggest that there is a need for migrant-focused family planning programmes. Specifically, family planning centres should be responsible for targeting migrants in the early years after their arrival in Cotonou. After some years of stay, migrants’ ability to space their births adequately would tend to converge with that of non-migrants in Cotonou. The intervention would then help to reduce risky sexual and reproductive behaviours among migrants upon arrival and accelerate the convergence. Interventions can also aim to improve access to family planning services for women living in rural areas and small cities and those who are more likely to migrate to large cities. Policies would be more efficient if they mainly focus on migrant women as well as women with low socioeconomic status. Moreover, interventions aimed at tailoring specific messages on the adverse health outcomes of long interbirth intervals should target non-migrant and earlier migrant women.

## Supplementary information

**Additional file 1.** Household questionnaire. This supplementary file is an MS Excel workbook that presents the household questionnaire.

**Additional file 2.** Women questionnaire. This supplementary file is an MS Excel workbook that presents the women questionnaire.

## Data Availability

The datasets used and analysed during the current study are available from the corresponding author on reasonable request.

## Abbreviations

CIF: Cumulative Incidence Function
EA: Enumeration Area
FMS: Fertility and Migration Survey
IBI: Interbirth Interval
ICF: Inner City Fund
INSAE: Benin Republic National Institute of Statistics
LMIC: Low- and Middle-Income Country
SHR: Subhazard Ratio
SSA: Sub-Saharan African

## Footnotes

1 The initial sample size (n) of 1963 households was based on an urban residence design effect (Deft) of 1.7, an estimated proportion of modern contraceptive use in Cotonou (p) of 12.5%, a desired relative standard error (RSE) of 0.1, a response rate of 94.7% for interviews with women aged 15–49 years in urban areas (R_i_), a household response rate in urban areas (R_h_) of 97.6%, and a desired number of eligible women per household (d) of 1.05. These figures are from the 2011–2012 Benin Republic Demographic and Health Survey (DHS) [12]. The formula for calculating this sample size is given by: $n={Deft}^2\times \frac{\left(1/p-1\right)}{RSE^2}/\left({R}_i\times {R}_h\times d\right)$. The 95% confidence interval calculated as *p* ± 2 ∗ *SE* is (0.085–0.165). A total of 1960 households were selected, of which 1913 occupied households were occupied. The number of completed interviews with women in this study (1949) is greater than the minimum (of 800) required by the MEASURE DHS program for high fertility countries [27].

2 Due to the sampling design used for the 2018 Cotonou FMS, sample weights are applied for the analysis in order to produce proper representations. This study’s sampling design follows DHS’s standard. And, as described in Rutstein and Rojas [27], the individual weight of a (woman) respondent’s case is the household weight multiplied by the inverse of the individual response rate of her individual response rate group.
